# Largely preserved vestibular function despite severe-to-profound hearing loss in Noonan syndrome spectrum disorders

**DOI:** 10.1007/s00405-026-10113-2

**Published:** 2026-03-20

**Authors:** Kento Koda, Kentaro Ichijo, Teru Kamogashira, Makoto Kinoshita, Mineko Oka, Kenji Kondo, Chisato Fujimoto

**Affiliations:** 1 Department of Otolaryngology and Head and Neck Surgery, The University of Tokyo, Hongo 7-3-1, Bunkyo-ku, Tokyo, 113-8655 Japan; 2 Department of Otolaryngology, Kanto Central Hospital, Federation of National Public Service Personnel Mutual Aid Associations, Tokyo, Japan

**Keywords:** Noonan syndrome spectrum disorders, Vestibular function, Hearing loss, Cochlear implantation, PTPN11

## Abstract

**Purpose:**

Noonan syndrome spectrum disorders (NSSDs) are RASopathies in which hearing impairment is common, whereas vestibular function has rarely been investigated systematically. This study aimed to evaluate auditory and vestibular function in patients with NSSDs and severe-to-profound hearing loss undergoing preoperative assessment for cochlear implantation.

**Methods:**

Three patients who were clinically diagnosed with Noonan syndrome and later genetically classified within the NSSDs framework underwent comprehensive auditory and vestibular evaluations, including auditory brainstem responses, behavioral audiometry, caloric testing, and cervical/ocular vestibular evoked myogenic potentials (cVEMPs and oVEMPs), when feasible for age and cooperation. Genetic testing identified pathogenic *PTPN11* variants.

**Results:**

All three patients had severe-to-profound hearing loss. Pathogenic PTPN11 variants were detected—p.Glu139Asp in two patients and p.Tyr279Cys in one, the latter typically associated with Noonan syndrome with multiple lentigines (NSML), although lentigines were absent due to young age. Vestibular function was largely preserved in one patient and mildly impaired in two patients. oVEMP testing was successfully completed in only one case, whereas it could not be completed in the two infant cases because of limited cooperation. Imaging studies revealed no inner ear malformations or enlargement of the vestibular aqueducts.

**Conclusion:**

In this case series of pediatric patients with PTPN11-related NSSDs and severe-to-profound hearing loss, vestibular function was largely preserved or only partially impaired. These findings demonstrate a dissociation between auditory and vestibular function and suggest that differential vulnerability to molecular signaling abnormalities among sensory organs may be involved.

**Supplementary Information:**

The online version contains supplementary material available at 10.1007/s00405-026-10113-2.

## Introduction

Noonan syndrome (NS) is a relatively common RASopathy, affecting approximately 1 in 1,000–2,500 live births [1]. It is characterized by distinctive craniofacial features, congenital heart disease, short stature, and variable developmental delay [2].

The Noonan syndrome spectrum disorders (NSSDs) are a group of related RASopathies that include NS, Noonan syndrome with multiple lentigines (NSML), cardio-facio-cutaneous syndrome (CFCS) and Costello syndrome (Table 1). Because their phenotypes frequently overlap, molecular testing is often required for definitive classification. The pathogenesis of NSSDs is largely attributed to germline variants in genes encoding components of the RAS/MAPK signaling pathway. NS is most frequently associated with pathogenic *PTPN11* variants [3, 4]. The *PTPN11* gene encodes the tyrosine phosphatase SHP2, a key regulator of this pathway [1, 5]. Similar variants also underlie a substantial portion of NSML cases [6].

**Table 1:** Overview of Noonan syndrome spectrum disorders (NSSDs ). NSSDs are part of the RAS/MAPK pathway–related genetic disorders (RASopathies), and include Noonan syndrome (NS), Noonan syndrome with multiple lentigines (NSML/LEOPARD syndrome), Costello syndrome (CS), and cardio-facio-cutaneous syndrome (CFCS). This study focuses on patients with NS and NSML. For conciseness, only the major causative genes for each condition are listed.

Disorder	Abbreviation	Major Causative Genes	Representative Mutations	Key Clinical Notes
Noonan syndrome	NS	PTPN11, SOS1, RAF1, RIT1, KRAS	PTPN11: p.Glu139Asp, p.Asp61Gly, p.Thr73Ile / SOS1: p.Thr266Lys / RAF1: p.Ser257Leu	Most common NSSDs; PTPN11 ≈ 50%
Noonan syndrome with multiple lentigines (NSML / LEOPARD)	NSML	PTPN11	PTPN11: *p*.Tyr279Cys, *p*.Thr468Met	Distinct phenotype with lentigines, hypertrophic cardiomyopathy
Cardio-facio-cutaneous syndrome	CFCS	*BRAF*, *MAP2K1*, *MAP2K2*, *KRAS*	BRAF: p.Val600Glu (V600E), p.Lys601Gln / MAP2K1: p.Pro124Arg / MAP2K2: p.Glu203Gln	Often more severe developmental delay
Costello syndrome	CS	*HRAS*	HRAS: p.Gly12Ser (G12S), p.Gly12Ala (G12A), p.Gly12Asp (G12D)	Coarse facies, tumor predisposition

Otorhinolaryngological manifestations are frequent in NSSDs. Hearing loss develops in 30–50% of patients due to middle ear or inner ear diseases [7]. Mutations in PTPN11 are the most frequent genetic cause of NS and have been associated primarily with conductive hearing loss, although cases of mixed and, more rarely, sensorineural hearing loss (SNHL) have also been reported [7, 8]. NSML and CFCS, in particular, show a high prevalence of SNHL [9, 10]. Furthermore, imaging studies focusing on auditory structures have revealed inner ear morphological abnormalities, such as a small dysplastic cochlea [11]. In patients with NSSDs who develop severe-to-profound sensorineural hearing loss, cochlear implantation may provide meaningful auditory benefit and is increasingly considered in clinical practice. Because cochlear implantation may affect vestibular function and vestibular dysfunction is known to influence postoperative balance and motor development [12, 13], preoperative evaluation for cochlear implantation represents a clinically relevant opportunity to assess vestibular function and establish the preoperative baseline in patients with NSSDs.

While auditory phenotypes have been relatively well studied, vestibular function in NSSDs remains poorly characterized. Only isolated reports describe patients with both SNHL and vestibular symptoms, and formal vestibular assessments are rarely documented [8]. Nevertheless, evidence suggests potential vestibular involvement in this patient group. Pathological studies have shown reduced spiral ganglion cells and vestibular structural anomalies [14]. Furthermore, the frequent motor developmental delay observed in NS raises the possibility that vestibular dysfunction contributes to these delays [8, 12, 15].

Although preoperative vestibular assessment is important in pediatric cochlear implant candidates for establishing a functional baseline and optimizing postoperative management [13], systematic studies addressing vestibular function in NSSDs are lacking. In this study, we analyzed a case series of three patients who were clinically diagnosed according to the criteria of van der Burgt [5]. All cases were subsequently genetically confirmed within the NSSDs framework, presenting with severe-to-profound SNHL and undergoing cochlear implant candidacy evaluation. A comprehensive vestibular test battery, including caloric testing and vestibular evoked myogenic potentials (VEMPs), was performed. This report represents a detailed case series to investigate vestibular function in this highly specific patient population.

## Patients and methods

### Patients

Patients who attended the vestibular function outpatient clinic at our department for preoperative evaluation of cochlear implantation between 2008 and 2025 were reviewed. Our institution is a tertiary referral center for hearing disorders, where a wide range of pediatric and adult patients with hearing loss are evaluated. Among these, all consecutive patients who received a definitive diagnosis of NSSDs (one adolescent and two infants) were included in the present study. No additional exclusion criteria were applied. This was a retrospective study conducted with the approval of the institutional ethics committee of the University of Tokyo Hospital (approval number 2487). All examinations had originally been performed as part of routine clinical assessments for cochlear implantation candidacy.

The diagnosis of NS was clinically established according to the clinical criteria proposed by van der Burgt et al., which are commonly applied in the initial evaluation of RASopathies. Genetic testing was subsequently performed in all patients for whom ethical approval and written informed consent were obtained. All patients who received a definitive molecular diagnosis within the NSSDs framework (one adolescent and two infants) were included in this study.

All patients underwent a comprehensive battery of audiological and vestibular tests, including auditory brainstem responses (ABR), behavioral audiometry, pure-tone audiometry (PTA), and speech discrimination testing, as well as vestibular assessments consisting of caloric testing, cervical and ocular VEMPs (cVEMPs and oVEMPs).

### Audiological evaluation

ABRs were recorded in a sound-treated booth using the Eclipse system (Interacoustics, Middelfart, Denmark). Click stimuli (90 dB nHL, rarefaction polarity, 21.1 Hz repetition rate) were delivered via insert earphones, and thresholds were defined as the lowest intensity level at which reproducible wave V responses could be identified.

Conditioned orientation response (COR) audiometry or play audiometry was performed in children to assess unaided bilateral hearing thresholds, using a clinical audiometer (AA-H1, Rion, Tokyo, Japan) in a sound-treated booth. PTA was performed in cooperative patients with the same audiometer, measuring air-conduction thresholds at 0.25, 0.5, 1, 2, and 4 kHz; bone-conduction thresholds were obtained when applicable. Speech discrimination testing was carried out with Japanese monosyllabic word lists (57-S test ), presented through the audiometer at each patient’s most comfortable listening level, and the percentage of correctly identified words was calculated for each ear [16].

### Genetic testing

Targeted resequencing analysis was performed using the NEXTFLEX Rapid XP system (PerkinElmer, Waltham, MA, USA), in combination with a NextSeq 500 or NextSeq 2000 next-generation sequencer (Illumina, San Diego, CA, USA). The panel included major RASopathy-associated genes such as PTPN11, SOS1, RAF1, RIT1, and KRAS. Variants were classified according to ClinVar and the 2015 ACMG/AMP guidelines [17].

### Caloric testing

Caloric responses were measured using standard electronystagmography (ENG; Interacoustics, Middelfart, Denmark). Ice water (4 °C, 2 mL) irrigation was performed as described previously [18]. Canal paresis > 20% or maximum slow-phase velocity < 10°/s was considered abnormal in adults, while age-specific normative values were applied for pediatric patients [19]. A response ≥ the age-adjusted lower normal limit was considered normal, < the limit was deemed decreased, and absence of nystagmus was considered negative.

### cVEMP

Air-conducted sound cervical vestibular evoked myogenic potentials (ACS cVEMP) testing was performed to assess the function of the saccule and inferior vestibular nerve, according to established clinical protocols [20, 21]. Recordings were obtained using the Eclipse system (Interacoustics, Middelfart, Denmark). Electromyography (EMG) activity was recorded from a surface electrode placed on the upper half of each sternocleidomastoid muscle (SCM). One ground electrode was placed on the forehead and one reference electrode on the sternal notch. Subjects were tested in a sitting position with the head rotated away from the side of stimulation, with SCM activity maintained between 20 and 200 µV RMS.

A 500-Hz tone burst (rise/fall time = 4 ms, plateau time = 2 ms, 100 dB nHL, equivalent to 123.5 dB pe-SPL) was presented through insert earphones. The first positive–negative peak (p13–n23) was identified, and latencies and peak-to-peak amplitudes were determined from the average of two runs, each consisting of 100 stimuli. Responses were normalized according to muscle activity. For amplitude evaluation, the asymmetry ratio (cVEMP AR) was calculated as 100 × [(Au − Aa) / (Aa + Au), where *Au* is the amplitude on the unaffected side and *Aa* is the amplitude on the affected side. where *Au* is the amplitude on the unaffected side and *Aa* is the x amplitude on the affected side. An absent response was defined as the absence of a reproducible p13–n23 in two runs. A decreased response was defined as a reproducible p13–n23 with an AR above this limit, indicating reduced function on the side with the smaller amplitude. Both an absent response and a decreased response were considered abnormal.

### oVEMP

Bone-conducted vibration ocular vestibular evoked myogenic potential (BCV oVEMP) testing was performed to evaluate the function of the utricle and superior vestibular nerve, based on established methodology [22, 23]. EMG electrodes were placed 1 cm below the center of each lower eyelid, with the ground electrode on the forehead and the reference electrode on the chin. Subjects were instructed to look upward approximately 20° above straight ahead.

Tone bursts (500 Hz, rise/fall time = 2 ms, plateau time = 2 ms, 70 dB nHL) were delivered at 5.1 Hz using a B81 bone-conduction transducer (Radioear, New Eagle, USA), secured with a steel spring headband and positioned symmetrically on the mastoid. One hundred responses were band-pass filtered (10–1,000 Hz), and averaged. Two runs were recorded for each ear to confirm reproducibility.

The N1–P1 peak-to-peak amplitude was measured, and raw amplitude values were analyzed.

The asymmetry ratio (oVEMP AR) was calculated as 100 × [(Au − Aa) / (Aa + Au), using signals recorded from the eye contralateral to stimulation. Based on normative data for the B81 transducer (mean ± SD = 23.1 ± 18.7) the normal upper limit for oVEMP AR was set at 60.5 (mean + 2SD) [24]. Based on normative data for the B81 transducer (mean ± SD = 23.1 ± 18.7) the normal upper limit for oVEMP AR was set at 60.5 (mean + 2SD) [24]. An absent response was defined as the absence of a reproducible N1–P1 in two runs. A decreased response was defined as a reproducible N1–P1 with an AR above this limit, indicating reduced function on the side with the smaller amplitude. Both an absent response and a decreased response were considered abnormal.

## Results

Three patients with NSSDs underwent comprehensive evaluation of auditory and vestibular function. The clinical and genetic features are summarized in Table 2, and the results of the auditory and vestibular assessments are presented in Tables 3 and 4, respectively. None of the patients exhibited inner ear malformations or enlargement of the vestibular aqueduct on imaging. Middle ear status was normal in all patients, with no evidence of effusion or conductive pathology on otoscopy and temporal bone computed tomography.

**Table 2:** Clinical characteristics and genetic findings of patients with NSSDs. This table summarizes age, sex, nucleotide and amino acid changes, and the pathogenicity of each PTPN11 variant identified in the three cases

Case	Age(year)	Sex	Nucleotide change	Amino Acid Change	Pathogenecity
Case 1	14	Male	c.417G > C (heterozygous)	p.Glu139Asp	Pathogenic
Case 2	1	Male	c.417G > C (heterozygous)	p.Glu139Asp	Pathogenic
Case 3	1	Female	c.836 A> G(heterozygous)	p.Tyr279Cys	Pathogenic

**Table 3:** Audiological evaluations of patients with NSSDs. Auditory findings include ABR results, unaided bilateral hearing thresholds obtained by COR/PLAY, pure-tone averages (PTA ), speech discrimination scores, onset and type of hearing loss, and age at which hearing devices were first fitted.(Abbreviations: ABR, auditory brainstem response; COR/PLAY, conditioned orientation reflex/play audiometry; PTA, pure-tone average; R, right; L, left )

Case	ABR	COR/PLAY(Bilateral unaided hearing)	PTA(*R*; Right/L; Left)	Speech discrimination test	Onset of hearing loss	Starting time for adding audible devices	Type of hearing loss at onset
Case 1	-	-	*R*; 98.8ངB L; 101.3ངB	*R*; 70dB 55% L; 70dB 30%	Congenital	1year old	Sensorineural (with minimal possible conductive component)
Case 2	100 dB unresponsive on both sides	111ངB	-	-	Congenital	6months old	Sensorineural
Case 3	R100dB L90dB	96.25dB	-	-	Congenital	4months old	Sensorineural

**Table 4:** Vestibular function test results in patients with NSSDs. Caloric responses, ACS cVEMP and BCV cVEMP findings, and ACS/BCV oVEMP results (categorized by eye side) are shown, along with the presence or absence of vestibular symptoms. (Abbreviations: ACS, air-conducted stimuli; BCV, bone-conducted vibration; cVEMP, cervical vestibular evoked myogenic potential; oVEMP, ocular vestibular evoked myogenic potential)

Case	Caloric test	ACS cVEMP	BCV oVEMP	Vestibular symptoms
Case 1	Left Canal Paresis 31.4%	Normal	Absent (Right)	No
Case 2	Normal	Decreased (Left)	-	No
Case 3	Normal	Normal	-	No

### Case 1 (14-year-old male)

This patient fulfilled the diagnostic criteria for NS, with major criteria of characteristic facial features and short stature and a minor criterion of learning disability. Neither parent exhibited relevant features, and no family history was identified, suggesting a sporadic case. Congenital hearing loss was managed with hearing aids from 1 year of age. Pure-tone thresholds were 98.8 dB (right) and 101.3 dB (left) with speech discrimination of 55% (right), and 30% (left) at 70 dB. Genetic analysis revealed a heterozygous PTPN11 variant c.417G > C (p.Glu139Asp), which is a pathognomonic mutation for NS. Vestibular testing showed left canal paresis of 31.4% in the caloric test. ACS cVEMPs were normal, whereas BCV oVEMPs were absent on the right. No vestibular symptoms were reported.

### Case 2 (1-year-old male)

Major criteria of characteristic facial features and short stature, together with minor criteria of cryptorchidism and patent foramen ovale, established the diagnosis of NS. No family history was identified, indicating a sporadic case. The patient presented with congenital hearing loss and started hearing aids at 6 months of age. ABR showed no response bilaterally at 100 dB, and COR/PLAY revealed a threshold of 111 dB. Pure-tone and speech testing were not feasible due to age. Genetic analysis confirmed the c.417G > C (p.Glu139Asp) variant, which is a pathognomonic mutation for NS. Vestibular evaluation showed normal caloric responses but a decreased ACS cVEMP response on the left. BCV oVEMP could not be completed due to limited cooperation. No vestibular symptoms were reported.

### Case 3 (1-year-old female)

Characteristic facial features and short stature met the major diagnostic criteria for NS. No family history was identified, suggesting a sporadic case. The patient began using hearing aids at 4 months of age. ABR showed responses at 100 dB (right) and 90 dB (left) with a COR/PLAY threshold of 96.25 dB. Pure-tone and speech testing were not performed.

Genetic testing revealed a heterozygous PTPN11 variant c.836 A > G (p.Tyr279Cys), which is a pathognomonic mutation for NSML. Although lentigines were not observed at this early age, the presence of this genotype indicates that the patient should be considered within the NSML spectrum despite the initial clinical diagnosis of NS.

Vestibular examinations showed normal caloric responses; ACS cVEMP and BCV oVEMP could not be completed because of age. No vestibular symptoms were observed.

### Summary

All patients were clinically suspected of NS based on characteristic facial features and subsequently received genetic confirmation within the NSSDs framework. All three carried pathogenic heterozygous PTPN11 variants and exhibited severe-to-profound hearing loss. The adolescent and one infant with the NS-associated c.417G > C (p.Glu139Asp) variant showed mild unilateral vestibular abnormalities without overt symptoms. The infant with the NSML-associated c.836 A > G (p.Tyr279Cys) variant showed largely preserved vestibular responses on objective testing despite severe-to-profound hearing loss.

## Discussion

This study represents a small case series of patients with NSSDs and is intended to be descriptive and hypothesis-generating rather than to draw definitive conclusions. In the two infant cases (Cases 2 and 3), oVEMP could not be evaluated; however, this does not indicate preserved or impaired utricular function but rather reflects technical limitations associated with age and limited cooperation. A key finding of our study was the dissociation between auditory and vestibular function. Despite having severe-to-profound congenital SNHL, the patients’ vestibular function was either unaffected or only mildly impaired.

In our series, two patients carried the pathogenic PTPN11 c.417G > C (p.Glu139Asp) variant, which has been reported predominantly in association with NS [6] and is considered a gain-of-function mutation [24], whereas one patient carried the pathogenic PTPN11 c.836 A > G (p.Tyr279Cys) variant, a well-established cause of NSML [25] with catalytically impaired (dominant-negative) effects [24]. Although the patient carrying the p.Tyr279Cys variant did not exhibit lentigines at the time of examination due to young age, this mutation is considered pathognomonic for NSML. Thus, our cohort included both activating and dominant-negative classes of PTPN11 variants.

From a biological perspective, gain-of-function variants associated with NS disrupt the autoinhibitory mechanism of SHP2, resulting in constitutively enhanced catalytic activity and predominantly leading to excessive activation of the RAS/MAPK pathway [5, 26]. This mechanism is consistent with experimental models suggesting that SHP2 activation can interfere with inner ear hair cell development and neural projections [27], and sustained MAPK activation has also been reported to be associated with increased oxidative stress and enhanced cellular vulnerability [26].

In contrast, dominant-negative variants associated with NSML abolish the catalytic activity of SHP2 while preserving its ability to bind to signaling complexes, leading to incomplete regulation of downstream signaling pathways [24, 28, 29]. This loss of function may result in a dual pathogenic mechanism. Specifically, reduced MAPK signaling may be insufficient to support normal inner ear development, while loss of SHP2 activity may cause relative hyperactivation of the PI3K/AKT/mTOR pathway, thereby increasing the vulnerability of sensory cells [30, 31]. Thus, although NS and NSML exert opposing effects on MAPK signaling, both conditions may ultimately lead to disruption of signaling balance within the cochlea, contributing to impairment of cochlear homeostasis and the pathogenesis of sensorineural hearing loss.

One possible explanation for the dissociation observed in our cohort—namely, pronounced cochlear dysfunction with relatively mild vestibular impairment—is differential vulnerability of the sensory epithelia. The cochlea, which requires highly precise spatiotemporal regulation to maintain tonotopic organization and metabolic homeostasis, may be more susceptible to disruption of signaling balance than the vestibular organs. In contrast, vestibular end organs have been reported to possess greater regenerative and compensatory capacity than the cochlea [32–34], and may therefore show a relatively greater capacity for functional recovery following signaling perturbation. Taken together, we hypothesize that in patients with PTPN11-related NSSDs, cochlear sensory epithelia are selectively vulnerable to signaling imbalance, whereas vestibular end organs are relatively preserved, resulting in a phenotype characterized by severe-to-profound sensorineural hearing loss with only mild vestibular dysfunction.

From a clinical perspective, vestibular function is closely linked to motor development [12], and cochlear implantation may carry a potential risk of postoperative vestibular dysfunction. Therefore, preoperative vestibular assessment may provide useful baseline information for interpreting postoperative balance outcomes and for counseling patients and families. Because the clinical phenotype of NSSDs is age-dependent, early diagnosis in infancy remains challenging. Characteristic facial features may be subtle at birth, and additional findings such as short stature, chest deformity, webbed neck, or electrocardiographic abnormalities tend to emerge progressively [35]. For example, the lentigines characteristic of NSML typically appear after four to five years of age [36]. Similarly, in NS, diagnostic features become more apparent with age [37]. Although genetic testing can aid diagnosis, practical limitations related to availability and informed consent remain. Thus, when physical findings useful for the diagnosis of NSSDs are not yet fully apparent in infancy, hearing loss may, in some cases, serve as a trigger for considering NSSDs.

This study is limited by the small sample size and the inability to perform genotype-specific comparisons. Whether the unilateral abnormalities observed in some cases are attributable to NS-related mechanisms or to individual anatomical variability also remains unclear. In addition, oVEMP testing could not be completed in two infant cases because of limited cooperation, which may have led to an underestimation of utricular dysfunction in these patients.

Nevertheless, these findings emphasize the importance of evaluating both auditory and vestibular function in patients with NSSDs. Larger, genotype-based studies are needed to clarify the auditory–vestibular phenotype associated with specific variants. Overall, our results highlight the need for integrating vestibular assessment into the routine otolaryngological evaluation of pediatric patients with suspected RASopathies.

## Conclusion

In this case series, all pediatric patients with NSSDs and severe-to-profound hearing loss were found to carry pathogenic PTPN11 variants, and their vestibular function was either preserved or only partially impaired. These findings demonstrate a dissociation between auditory and vestibular function in NSSDs, suggesting that differential vulnerability to molecular signaling abnormalities among sensory organs may be involved.

## Supplementary Information

Below is the link to the electronic supplementary material.

Supplementary Material 1

## Data Availability

The raw data supporting the conclusions of this article will be made available by the authors, without undue reservation.
