# Supplementary material for: Largely preserved vestibular function despite severe-to-profound hearing loss in Noonan syndrome spectrum disorders
Source: Eur Arch Otorhinolaryngol. 2026 Mar 20;283(6):3725–32. doi: 10.1007/s00405-026-10113-2 (PMC13249677; doi:10.1007/s00405-026-10113-2)
Supplement: Supplementary file 1 — Supplementary Material 1 [file 405_2026_10113_MOESM1_ESM.docx]

**Revised Manuscript Submission – EAOR-D-26-00213**

**Dear Prof. Bernal-Sprekelsen,**

Thank you very much for your letter regarding our manuscript entitled
“Preserved Vestibular Function Despite Severe Hearing Loss in Noonan Syndrome Spectrum Disorders” (Ms. No. EAOR-D-26-00213), submitted to *European Archives of Oto-Rhino-Laryngology*.

We sincerely appreciate the time and effort you and the reviewers have devoted to evaluating our work. We are grateful for the constructive and insightful comments, which have helped us to improve the clarity and scientific rigor of the manuscript.

In response to the reviewers’ suggestions, we have carefully revised the manuscript. All changes have been clearly marked in red in the revised version. In addition, we have provided a detailed point-by-point response to each reviewer’s comment at the beginning of the manuscript, including specific page and line references to facilitate evaluation.

We believe that the revisions have strengthened the manuscript and clarified several important methodological and interpretative aspects. We hope that the revised version will now be suitable for publication in *European Archives of Oto-Rhino-Laryngology*.

Thank you again for your consideration. We look forward to your further review.

Yours sincerely,

**Sincerely,**

Chisato Fujimoto, MD, PhD
Department of Otolaryngology–Head and Neck Surgery
The University of Tokyo Hospital
Tokyo, Japan
Email: cfujimoto-tky@umin.ac.jp
